# Negative life events and depression by gender in the Brazilian Longitudinal Study of Adult Health (ELSA-Brazil)

**DOI:** 10.1016/j.clinsp.2024.100488

**Published:** 2024-10-15

**Authors:** Simone V. Silva, Itamar S. Santos, Danielle B. Lima, Alessandra C. Goulart, Ana C. Varella, Paulo A. Lotufo, Andre R. Brunoni, Isabela M. Bensenor

**Affiliations:** a Faculdade de Medicina, Universidade de São Paulo (FMUSP), São Paulo, SP, Brazil; b Centro de Pesquisa Clínica e Epidemiológica, Hospital Universitário, Universidade de São Paulo (HUUSP), São Paulo, SP, Brazil; c Departamento de Saúde Pública, Faculdade de Ciências Médicas, Santa Casa de São Paulo (FCMSCSP), São Paulo, SP, Brazil; d Serviço Interdisciplinar de Neuromodulação, Laboratório de Neurociências (LIM-27), Instituto de Psiquiatria, Hospital das Clínicas, Faculdade de Medicina, Universidade de São Paulo (HCFMUSP), São Paulo, SP, Brazil; e Serviço de Eletroconvulsoterapia, Instituto de Psiquiatria, Hospital das Clínicas, Faculdade de Medicina, Universidade de São Paulo (HCFMUSP), São Paulo, SP, Brazil

**Keywords:** Negative Life Events, Stressful Life Events, Stress, Prevalent Depression, Incident Depression, Gender

## Abstract

**Objective:**

Gender differences may interfere with the association between Negative Life Events (NLEs) and prevalent/incident depression. This study evaluated the effect of gender in this association using data from the ELSA-Brazil cohort.

**Methods:**

The authors analyzed 15,088 participants (mean age, 52.1 (9.1), 54.4 % women). NLEs (robbery, hospitalization, death of a close relative, financial hardship, and rupture of a love relationship) were accessed at baseline. Depression was assessed at baseline and follow-ups. The authors built logistic (Odds Ratio [OR], 95 % Confidence Interval [95 % CI]) and Poisson regression models (Relative Risk [RR], [95 % CI]) to evaluate this association.

**Results:**

Women reported more NLEs compared to men. The authors found associations between NLEs and prevalent depression: for men, hospitalization (OR = 1.83; 95 % CI 1.16‒2.91), financial hardship (OR = 2.42; 95 % CI 1.69‒3.49), rupture of a love relationship (OR = 2.54; 95 % CI 1.50‒4.29), and any NLE (OR = 2.30; 95 % CI 1.59‒3.35); and for women, robbery (OR = 1.81; 95 % CI 1.31‒2.49), hospitalization (OR = 1.46; 95 % CI 1.11‒1.92), financial hardship (OR = 1.76; 95 % CI 1.43‒2.17), rupture of a love relationship (OR = 1.66; 95 % CI 1.20‒2.32), and any NLE (OR = 1.65; 95 % CI 1.34‒2.04). For incident depression only financial hardship (RR = 2.09; 95 % CI 1.55‒2.83) was associated with depression in men, while, for women, robbery (RR = 1.54; 95 % CI 1.16‒2.04); hospitalization (RR = 1.36; 95 % CI 1.07‒1.74), financial hardship (RR = 1.37; 95 % CI 1.14‒1.65), and any NLE (RR = 1.25; 95 % CI 1.04‒1.49) were associated with incident depression. No association was found between the death of a close relative and prevalent/incident depression.

**Conclusion:**

NLEs were associated with depression in men and women, with a higher impact in the latter.

## Highlights

• NLEs were associated with prevalent depression in men and women.
• NLEs were associated to incident depression in women
• Only financial hardship was associated to incident depression in men.
• LIMITATIONS
• NLEs questionnaire was self-reported with possible memory bias.
• Depression was defined by validated scale but some misclassification is possible.

## Introduction

Negative Life Events (NLEs) are stressors that may affect daily life individually and socially such as robbery or any kind of aggression, health problems, loss of a close relative or friend, financial problems, or end of a love relationship.[1], [2], [3] Negative life events are associated with the course and severity of various mental disorders.^4^^,^^5^ In Brazil, negative life events were previously studied using a questionnaire validated and adapted to Brazilian Portuguese for civil servants in the Pro-Saude study and also in the ELSA-Brasil.^6^^,^^7^

Between 1990 and 2019 the number of cases of mental disorders increased by 48.1 %. In addition, 80.6 % of the burden associated with mental disorders occurred among individuals between 16 and 65 years of age. Despite evidence-based interventions, there is no evidence of a global reduction in this burden since 1990.^8^ Depression is a mental disorder closely associated with reduced life expectancy and is an important risk factor for chronic diseases and suicide.[8], [9], [10], [11] Depressive disorders are among the leading causes of disability worldwide, ranking 13^th^ among the leading causes of increases in Disability Adjusted Life Years (DALYs).^8^^,^^12^ Depression is more frequent in women compared to men.^13^ Therefore, the effect of NLEs may impact women and men in different ways.

Some studies indicate that men are more vulnerable to negative life events compared to women.^14^^,^^15^ However, most of the studies report that women are more exposed to negative life events and develop depression more frequently than men following a negative life event.^2^^,^^16^^,^^17^ The results of some studies show no differences between the genders in the effect of negative life events in depression for financial difficulties, death of a spouse or child, marital problems or divorce, legal problems, disease, and violence.^1^^,^^2^ Other results report that, in women, more distant interpersonal relationships losses, such as the death of a close friend or relative; change of residence; physical attack or life-threatening illness/injury; loss of spouse, child, or parent; relationship problems; and family dysfunctions are associated with depression.^1^^,^^2^^,^^3^^,^^18^ In men, there are findings that indicate that divorce, job loss, legal problems, robbery, and work problems are the negative life events associated with depression.^2^^,^^18^ Therefore, there are conflicting data about the association between negative life events and depression according to gender. Some authors suggest that differences in the association between negative life events and depression according to gender must be more deeply explored using prospective studies.^16^^,^^19^

The purpose of this article is to investigate, both cross-sectionally and prospectively, the association between negative life events that occurred within the previous twelve-month period and the prevalence of depression at baseline and the incidence of depression at 4-year and 8-year follow-ups according to gender, using data from the Brazilian Longitudinal Study of Adult Health (ELSA-Brazil), a prospective cohort study of a highly diverse population, previously unexplored in a low-middle-income country like Brazil.

## Materials and methods

The ELSA-Brazil study is a multicenter prospective cohort study that aims to investigate the relationship between sociodemographic, clinical, psychosocial, and lifestyle factors and the development of cardiovascular diseases and diabetes in a sample of 15,105 civil servants from six research universities and research institutions located in six Brazilian state capitals. At baseline (2008‒2010), the inclusion criteria were being an active or retired employee of the six institutions and aged between 35 and 74 years. Exclusion criteria were the presence of severe communication or cognitive problems, being pregnant or having given birth less than four months before joining the study sample, intending to leave work in the near future, and residing outside the metropolitan area where the Research Centers were located.[20], [21], [22]

The study had a 4-year follow-up (2012‒2014) and an 8-year follow-up (2017‒2019). The baseline and the follow-ups included comprehensive interviews, which addressed information on sociodemographic, clinical, and psychosocial characteristics, mental health, and laboratory tests. The same standard study protocol established procedures that were performed identically at all six investigation centers. All procedures were performed under strict supervision to ensure excellent quality control.[20], [21], [22] The ELSA-Brazil study followed internationally established ethical standards.^18^ The participants included in the study provided their informed consent by signing a consent form that was approved by the Research Ethics Committee of the six institutions involved. More information about ELSA-Brazil is provided elsewhere.[20], [21], [22]

**Fig. 1:**
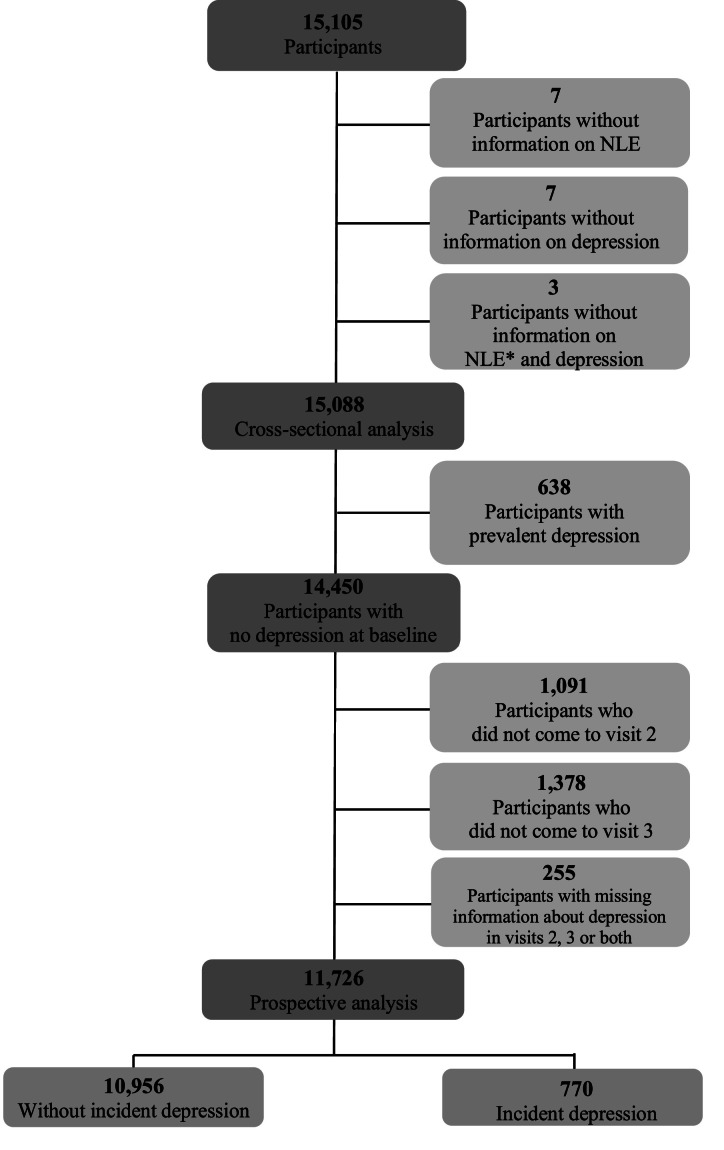
Sample flowchart. *NLE, negative life event.

In the present analysis, the authors excluded 17 of the 15,105 original study participants: 7 with no information about depression, 7 with no information about negative life events, and 3 with no information about depression and negative life events. Among the remaining 15,088 participants in the cross-sectional analysis, there were 638 (4.2 %) cases of prevalent depression and 14,450 (95.8 %) participants without depression. In the prospective analysis, all 638 cases of prevalent depression were excluded from the analysis. The authors also excluded 2,724 participants who did not come either to the 4-year follow-up (n = 1,091) or to the 8-year follow-up (n = 1378), or who were missing incident depression information (n = 255), resulting 11,726 participants in the prospective analysis, 770 (6.6 %) with incident depression at the 4-year or 8-year follow-up and 10,956 (93.4 %) without depression (Fig. 1).

### Negative life events and depression

Life events in ELSA-Brasil were assessed using a structured questionnaire validated and adapted to Brazilian Portuguese including the more commonly cited negative life events.^6^^,^^7^ The five questions included in the questionnaire are: (a) “In the past 12 months, were you robbed, that is, had money or any goods taken by the use or threat of violence, or physical aggression?”; (b) “In the last 12 months, were you hospitalized for one night or more, due to illness or accident (except childbirth)?”; (c) “In the last 12 months, did a close relative (parent, spouse, partner, child or sibling) die?”; (d) “In the last 12 months, did you face a financial hardship more serious than usual?”; and (e) “In the last 12 months, did you suffer any disruption of a loving relationship, including divorce or separation?”.^7^ In this analysis, results were presented for each type of life event and also for any life event, with all negative life events combined in a dichotomous variable (yes/no).

Depression in the ELSA-Brazil study was assessed using the Portuguese version of the Clinical Interview Schedule Revised ‒ CIS-R.^23^^,^^24^ The CIS-R is a structured interview designed to quantify and diagnose non-psychotic psychiatric morbidity in the general population. It consists of a list of 14 relevant symptoms, including somatic complaints, fatigue, concentration and memory difficulties, sleep disturbances, irritability, physical health concerns, depressive manifestations, depressive ideation, excessive worry, anxiety, phobias, panic attacks, compulsions, and obsessions, using a 7-day reference period. Through the judicious combination of identified symptoms, the CIS-R provides diagnoses based on the International Statistical Classification of Diseases and Related Health Problems (ICD-10).^25^ In this study, the authors analyzed the occurrence of depressive disorder (F32) at baseline and at 4-year and 8-year follow-ups. Prevalent depression was verified at baseline (2008‒2010). Incident depression was defined as the emergence of depression in participants who did not present it at the baseline evaluation.

### Variables of interest

The variables included in the study were age (continuous); gender (man/woman*); self-reported race, defined according to the Brazilian Census classification (White, Mixed, Black, Asian, Indigenous); education attainment (less than high school, high school and some college, and complete college or more); average net monthly family income in US$ (less than 1,245; 1,245‒3,320; or > 3,320; local currency: US$ 1 = R$ 2); marital status (single/not single); living with someone (yes/no); have children (yes/no); being a caregiver (yes/no); attendance at religious activities (yes/no); self-perception of health (very good, good, regular, bad, very bad); smoking (never/past/current); alcohol intake (never/past/current); physical activity (inactive/insufficiently active/active); depressive disorder (yes/no); generalized anxiety disorders (yes/no); hypertension (yes/no); diabetes (yes/no); dyslipidemia (yes/no). The presence of comorbidities was defined as none, 1, or 2 or more of the following diseases/risk factors: coronary heart disease, stroke, hypertension, diabetes, dyslipidemia, asthma, chronic obstructive pulmonary disease, rheumatic diseases (arthritis, lupus erythematosus, and other types of arthritis), cirrhosis of the liver or chronic hepatitis, cancer, and obesity. The choice of these variables was based on the most common chronic diseases/risk factors in the study sample. Participants were also asked about the use of antidepressant medication in the last 2 weeks.

### Statistical analysis

Categorical variables are presented as absolute numbers and percentages and were compared using the Chi-Square test. Continuous variables are presented as means and Standard Deviations (SD) and were compared using Analysis of Variance (ANOVA) if they were normally distributed and the median (interquartile range) if they were not normally distributed. The normality of the variables was evaluated by the Kolmogorov-Smirnov test.

For each negative life event in the cross-sectional analysis, the Odds Ratio (OR) and 95 % Confidence Interval (95 % CI) for the association between any and each negative life event and depression were calculated and presented with no adjustment, adjusted for sociodemographic risk factors (age, education, and race – Model 1), and with multivariate adjustment for other confounding factors (Model 1 plus attendance at religious services, living with another person, self-perception of health, presence of comorbidities, and the use of antidepressant medication). All analyses were presented according to sex.

In the prospective analysis, the Relative Risk (RR) (95 % CI) was calculated using Poisson regression and presented with the same adjustments as in the cross-sectional analysis. A significance level of 0.05 was considered. The analyses were performed using the Statistical Package for Social Sciences (SPSS) version 25.0 software.^26^

## Results

The frequency of any negative life event was 39.7 % for men and 44.8 % for women (p < 0.0001). The frequencies of financial hardship, hospitalization, and rupture of a love relationship were higher in women, 23.2 %, 10.1 %, 6.51 %, respectively, as compared to men at 18.4 %, 9.1 %, and 5.6 % (p < 0.0001, p < 0.0001, and p = 0.005, respectively). There were no differences in the occurrence of robbery or death of a close relative between men and women. The frequency of depression was 5.8 % for women and 2.3 % for men (p < 0.0001) at baseline. Frequency of depression at follow-up was 5.2 % for women and 2 % for men (p < 0.0001).

Table 1 describes the general and clinical characteristics of the sample included in the cross-sectional analysis according to gender. The mean age of the participants was 52.1 years old (SD = 9.1) and 54.4 % were women. The frequency of self-reported race as White or Mixed was lower and as Black was higher, in women compared to men (p < 0.0001), while income was lower (p < 0.0001) and education attainment was higher (p < 0.0001) in women compared to men. Women reported a higher frequency of self-reported perception of health as very good and a better profile of smoking and alcohol intake, but more physical inactivity as compared to men. Men reported a higher frequency of living with someone and having children while women reported a higher frequency of attendance at religious services and of being a caregiver. More men than women were diagnosed with hypertension and diabetes. The frequency of generalized anxiety disorder, comorbidities, and the use of antidepressants was higher in women compared to men.

**Table 1:** General characteristics of participants at baseline by gender.

Gender			
General characteristics	Men (n = 6,880)	Women (n = 8,208)	p-value
Age in years, mean (SD)	52.2 (9.3)	52 (8.9)	0.36
Self-reported race (%)			<0.0001
White	3,597 (53)	4,188 (51.5)	
Mixed	2,026 (29.9)	2,174 (23.8)	
Black	941 (13.9)	1,455 (17.9)	
Asian	128 (1.9)	245 (3)	
Indigenous	92 (1.4)	65 (0.8)	
Education attainment (%)			<0.0001
Less than high school	1,138 (16.5)	792 (9.5)	
High school and some college	2,268 (33)	2,960 (36.1)	
Complete college or more	3,474 (50.5)	4,466 (54.4)	
Body mass index (BMI (kg/m^2^) (SD)	27 (4.3)	27.1 (5.1)	0.24
Income (US$) (%)			<0.0001
Up to 1,245	1,810 (26.4)	2,181 (26.7)	
1,245‒3,319	2,455 (35.8)	3,251 (39.8)	
≥3,320	3,590 (37.8)	2,741 (33.5)	
Self-reported health status (%)			<0.0001
Very good	1,807 (26.3)	2,415 (29.4)	
Good	3,700 (53.8)	4,157 (50.7)	
Regular	1,260 (18.3)	1,447 (17.6)	
Bad	93 (1.4)	146 (1.8)	
Very bad	16 (0.2)	42 (0.5)	
Smoking (%)			<0.0001
Never	3,463 (50.3)	5,121 (62.4)	
Past	2,433 (35.4)	2,095 (25.5)	
Current	983 (14.3)	922 (12.1)	
Alcohol intake (%)			<0.0001
Never	317 (4.6)	1,296 (15.8)	
Past	1,361 (19.8)	1,673 (20.4)	
Current	5,198 (75.6)	5,226 (63.8)	
Physical activity at leisure (%)			<0.0001
Inactive	4,979 (73.4)	6,466 (80)	
Insufficiently active	1,046 (15.4)	1,028 (12.7)	
Active	758 (11.2)	592 (7.3)	
No single (%)	5,626 (81.8)	4,347 (53)	<0.0001
Living with someone (%)	6,186 (89.9)	7,084 (86.3)	<0.0001
Have children (%)	5,848 (85)	5,282 (76.5)	<0.0001
Being caregiver (%)	580 (8.4)	879 (10.7)	<0.0001
Religious service attendance (%)	4,250 (61.8)	6,218 (75.8)	<0.0001
Hypertension (%)	2,756 (40.1)	2,636 (32.1)	<0.0001
Diabetes (%)	1,393 (20.3)	1,207 (14.7)	<0.0001
Dyslipidemia (%)	3,203 (46.7)	3,719 (45.4)	0.12
Generalized anxiety disorder (%)	591 (8.7)	1,376 (16.9)	<0.0001
Depressive disorder (%)	159 (2.3)	479 (5.8)	<0.0001
Use of antidepressant (%)	576 (11)	1,646 (24.9)	<0.0001
Comorbidities (%)			<0.0001
0	1,415 (20.7)	1,838 (22.6)	
1	5,006 (73.4)	5,728 (70.3)	
2 or more	399 (5.9)	577 (7.1)	
Robbery (%)	442 (6.4)	552 (6.7)	0.46
Hospitalization (%)	626 (9.1)	828 (10.1)	0.04
Death of a close relative (%)	743 (10.8)	944 (11.5)	0.17
Financial hardship (%)	1,265 (18.4)	1,905 (23.2)	<0.0001
Rupture of a love relationship (%)	388 (5.6)	555 (6.8)	0.005
Any negative life event (%)	2,729 (39.7)	3,671 (44.7)	<0.0001

Table 2 shows the findings of the logistic models for men and women. For men, after multivariable adjustment, hospitalization (OR = 1.83; 95 % CI 1.16‒2.91), financial hardship (OR = 2.42; 95 % CI 1.69‒3.49), rupture of a love relationship (OR = 2.54; 95 % CI 1.50‒4.29), and any negative life event (OR = 2.30; 95 % CI 1.59‒3.35) were associated with prevalent depression. No association with prevalent depression was found for robbery and the death of a close relative in men. For women, after multivariate adjustment, robbery (OR = 1.81; 95 % CI 1.31‒2.49), hospitalization (OR = 1.46; 95 % CI 1.11‒1.92), financial hardship (OR = 1.76; 95 % CI 1.43‒2.17), rupture of a love relationship (OR = 1.66; 95 % CI 1.20‒2.32), and any negative life event (OR = 1.65; 95 % CI 1.34‒2.04) were associated with prevalent depression. No association with prevalent depression was found for the death of a close relative for either men or women.

**Table 2:** Odds Ratio (OR) and 95 % Confidence Interval (95 % CI) of the association between each negative life event and depression at baseline.

Logistic models			
Men			
Negative life events	Crude	Adjusted by age, education, and race	Multivariable model^a^
n = 6,880	1.0 (Reference)	1.0 (Reference)	1.0 (Reference)
Robbery with threat or physical aggression	1.31 (0.73‒2.32)	1.35 (0.76‒2.41)	0.95 (0.50‒1.81)
Hospitalization	2.49 (1.67‒3.73)	2.58 (1.71‒3.89)	1.83 (1.16‒2.91)
Death of a close relative	1.19 (0.74‒1.92)	1.12 (0.69‒1.82)	1.05 (0.63‒1.77)
Financial hardship	3.84 (2.79‒5.27)	3.73 (2.68‒5.20)	2.42 (1.69‒3.49)
Rupture of a love relationship	3.60 (2.35‒5.53)	3.48 (2.25‒5.36)	2.54 (1.50‒4.29)
Any negative life event	3.52 (2.50‒4.94)	3.42 (2.42‒4.82)	2.30 (1.59‒3.35)
**Women**			
n = 8,208	1.0 (Reference)	1.0 (Reference)	1.0 (Reference)
Robbery with threat or physical aggression	2.11 (1.58‒2.80)	2.18 (1.63‒2.91)	1.81 (1.31‒2.49)
Hospitalization	1.97 (1.53‒2.52)	1.86 (1.44‒2.39)	1.46 (1.11‒1.92)
Death of a close relative	0.76 (0.71‒1.28)	0.95 (0.71‒1.28)	0.93 (0.68‒1.27)
Financial hardship	2.83 (2.35‒3.42)	2.55 (2.10‒3.09)	1.76 (1.43‒2.17)
Rupture of a love relationship	2.09 (1.57‒2.78)	2.04 (1.52‒2.73)	1.66 (1.20‒2.32)
Any negative life event	2.41 (1.99‒2.92)	2.24 (1.84‒2.72)	1.65 (1.34‒2.04)
**All**			
n = 15,088	1.0 (Reference)	1.0 (Reference)	1.0 (Reference)
Robbery with threat or physical aggression	1.90 (1.47‒2.44)	1.94 (1.50‒2.51)	1.56 (1.18‒2.08)
Hospitalization	2.13 (1.73‒2.63)	2.02 (1.63‒2.50)	1.57 (1.24‒1.99)
Death of a close relative	1.03 (0.80‒1.32)	0.99 (0.77‒1.28)	0.97 (0.74‒1.27)
Financial hardship	3.22 (2.74‒3.78)	2.80 (2.37‒3.31)	1.90 (1.58‒2.28)
Rupture of a love relationship	2.51 (1.98‒3.18)	2.36 (1.85‒3.01)	1.91 (1.45‒2.52)
Any negative life event	2.75 (2.33‒3.25)	2.49 (2.10‒2.95)	2.05 (1.72‒2.44)

a Adjusted by age, education attainment, self-reported race, marital status, living with someone, having children, attendance at religious activities, working as caregiver, self-reported health status, smoking, alcohol intake, physical activity at leisure, depressive disorder, general anxiety disorder, hypertension, diabetes, dyslipidemia, having comorbidities, and antidepressant use.

Table 3 describes the relative risk after multivariable adjustment for men and women. For men, only one negative life event was associated with incident depression: financial hardship (RR=2.09; 95 % CI 1.55‒2.83, p = 0.001). For women, robbery (RR = 1.54; 95 % CI 1.16‒2.04); hospitalization (RR = 1.36; 95 % CI 1.07‒1.74); financial hardship (RR = 1.37; 95 % CI 1.14‒1.65); and any negative life event (RR = 1.25; 95 % CI 1.04‒1.49) were associated with incident depression. No association was found between the death of a close relative and incident depression for men or women.

**Table 3:** Relative Risk (RR) and 95 % Confidence Interval (95 % CI) of the association between each negative life event and incidence of depression at 4-year and 8-year follow-ups.

Poisson regression			
Men			
Negative life events	Crude	Adjusted by age, education, and race	Multivariable model^a^
n = 5,311	1.0 (Reference)	1.0 (Reference)	1.0 (Reference)
Robbery with threat or physical aggression	1.70 (1.11‒2.60)	1.62 (1.05‒2.50)	1.43 (0.89‒2.32)
Hospitalization	1.79 (1.23‒2.60)	1.95 (1.34‒2.85)	1.25 (0.77‒2.03)
Death of a close relative	1.40 (0.92‒2.13)	1.47 (1.00‒2.14)	1.40 (0.92‒2.13)
Financial hardship	3.18 (2.44‒4.15)	2.69 (2.05‒3.53)	2.09 (1.55‒2.83)
Rupture of a love relationship	1.87 (1.20‒2.92)	1.67 (1.06‒2.63)	1.19 (0.69‒2.07)
Any negative life event	2.78 (2.12‒3.66)	2.56 (1.95‒3.37)	1.95 (1.44‒2.64)
**Women**			
n = 6,415	1.0 (Reference)	1.0 (Reference)	1.0 (Reference)
Robbery with threat or physical aggression	1.70 (1.32‒2.19)	1.81 (1.41‒2.33)	1.54 (1.16‒2.04)
Hospitalization	1.70 (1.37‒2.11)	1.69 (1.36‒2.11)	1.36 (1.07‒1.74)
Death of a close relative	1.14 (0.90‒1.45)	1.10 (0.87‒1.40)	1.08 (0.85‒1.38)
Financial hardship	1.96 (1.66‒2.31)	1.76 (1.49‒2.09)	1.37 (1.14‒1.65)
Rupture of a love relationship	1.50 (1.15‒1.95)	1.53 (1.18‒1.99)	1.13 (0.83‒1.52)
Any negative life event	1.67 (1.42‒1.96)	1,58 (1.35‒1.86)	1.25 (1.04‒1.49)
**All**			
n = 11,726	1.0 (Reference)	1.0 (Reference)	1.0 (Reference)
Robbery with threat or physical aggression	1.69 (1.36‒2.11)	1.74 (1.40‒2.17)	1.49 (1.17‒1.89)
Hospitalization	1.75 (1.45‒2.12)	1.77 (1.46‒2.14)	1.34 (1.08‒1.67)
Death of a close relative	1.25 (1.03‒1.53)	1.22 (1.00‒1.49)	1.14 (0.93‒1.41)
Financial hardship	2.33 (2.03‒2.69)	2,09 (1,80‒2.42)	1.58 (1.35‒1.86)
Rupture of a love relationship	1.66 (1.32‒2.08)	1.66 (1.32‒2.08)	1.09 (0.84‒1.43)
Any negative life event	1.98 (1.73‒2.28)	1.87‒1.62‒2.15)	1.41 (1.21‒1.65)

a Adjusted by age, education attainment, self-reported race, marital status, living with someone, having children, attendance at religious activities, working as caregiver, self-reported health status, smoking, alcohol intake, physical activity at leisure, depressive disorder, general anxiety disorder, hypertension, diabetes, dyslipidemia, having comorbidities, and antidepressant use.

## Discussion

The association between negative life events and prevalent depression was similar in women compared to men. The unique difference was the association of robbery or any type of aggression with depression in women but not in men. For incident depression in men, only financial hardship was associated with depression, while, for women, robbery, hospitalization, and financial hardship were associated with incident depression. Death of a close relative was not associated with either prevalent depression or incident depression in men or in women. Financial hardship was the only negative life event associated with prevalent and incident depression for both men and women.

An Italian cross-sectional analysis with 626 depressed patients (mean age, 55.1; SD = 16.1; 52 % women) showed a higher frequency of stressful life events in the last 6 months in women compared to men.^27^ In a cross-sectional study with 8,832 participants (53 % women, ranging in age from 18‒64 years), women reported more negative life events than men (63 % and 58 %, respectively, p < 0.03).^2^ The present results also found a higher number of negative life events in women as compared to men for all reported events.

Some cross-sectional studies show differences in the profile of negative life events between men and women. Life events such as aggression, serious problems with friends/relatives, and major financial crises show a significant association with depression for both; but divorce due to marital problems, unemployment, and problems with the police or the court are only significantly associated with depression in men; whereas the death of a parent/child/spouse is associated with depression in women.^2^ A study in the United States showed that domestic and sexual violence was associated with depression in women while having been in combat, being robbed, and exposed to toxins were more associated with depression in men.^28^ A large Chinese study reported a higher association between family-related events and depression in women and a higher association between finance-related events and depression in men.^3^

The unique difference in the profile of negative life events between women and men in the cross-sectional analysis was that robbery or aggression was only associated with depression in women. This is in accordance with a previous study ^28^ that reported the greater vulnerability of women to various types of violence but in discordance with Gonggrijp et al.^29^ who did not find any relationship between violence and depression in women. In this sample, financial hardship was associated with prevalent depression in men and women, as shown in previous analyses.^2^^,^^16^ However, familial negative life events, such as the death of a close relative, were not associated with depression either for women or for men, which disagrees with previous studies, especially for women.^28^^,^^30^^,^^31^

Regarding the strength of the association, an Iranian study with 4,763 participants reported that personal and social stressors showed a stronger association in women compared to men.^16^ A Chinese cross-sectional study with 8,711 elderly subjects (mean age 69.1; SD = 7.5) with very low education attainment, reported an OR = 1.6 times stronger in women compared to men for prevalent depression symptoms.^17^ Dalgard et al. (2006) ^2^ also reported similar results with stronger association in women compared to men. In the study results, the authors did not find great differences in the strength of association between negative life events and prevalent depression according to gender. However, this data agrees with previous findings that did not find any differences regarding the strength of the association of negative life events between men and women).^15^^,^^18^^,^^19^

Few longitudinal studies evaluate the relationship between negative life events and depression and the results are heterogeneous. A retrospective longitudinal study with 3 years of follow-up and a community sample of 2,824 participants (1,024 men and 1,800 women) evaluated the probability of developing depressive episodes in response to stressful events and concluded that women were three times more likely than men to suffer from severe depression in response to any stressful event. There was no difference between men and women in the risk of depression associated with the death of a spouse or child (close interpersonal relationships), divorce and marital problems, or acute financial or legal difficulties. Women were at increased risk of depression associated with the death of a close friend or relative (more distant interpersonal relationships), moving house, physical attack, or life-threatening illness.^1^ A longitudinal study with 32,744 participants and a mean follow-up of 3 years (mean age = 45.4 years, 63.9 % married) showed a stronger association between negative life events and depression in men compared to women.^32^ Another American longitudinal study, with a sample of 1,129 individuals who were followed for 25 years, also reported a stronger association of negative life events in men compared to women.^14^ The longitudinal results showed significant differences in the association between negative life events and incident depression between men and women. For men, only financial hardship was associated with incident depression, while for women, except for the death of a close relative and the rupture of a love relationship, all other negative life events were associated with incident depression. Therefore, the impact of negative life events in the analysis of the incidence of depression was much more significant in women compared to men.

Considering that individuals who experience negative life events are twice as likely to develop depression, with an earlier onset and more frequent, recurrent, and prolonged depressive episodes, as well as a worse response to antidepressants and a greater risk of attempting suicide,^4^^,^^15^ it is important to understand the mechanisms by which negative life events contribute to the onset and course of depression in each gender.^33^^,^^34^ The vast majority of studies suggest that women are more sensitive than men to the effects of negative events and develop more severe depression,[1], [2], [3]^,^^16^^,^^17^^,^^27^^,^^28^ possibly associated with hormonal fluctuations during the reproductive years.^35^ Hormonal fluctuations may modulate women's susceptibility to stress, brain functions, and inflammatory activity and reactivity, contributing to an increased risk of developing depressed moods related to inflammation and other neuropsychiatric, neurodevelopmental, and neurodegenerative disorders.[35], [36], [37], [38] However, the exact mechanism involved in this association is not clear. It is also important to clarify the possible risk factors that reported an association between negative life events and depression in men, suggesting that other mechanisms beyond hormone levels may also be investigated.^37^^,^^39^

ELSA-Brazil is a prospective cohort, with a multiethnic sample in a middle-income country, that makes it possible to analyze the association between negative life events and depression in both cross-sectional and longitudinal analyses showing important differences according to gender. The study includes many variables that can measure social support that was included as part of the adjustment in multivariable models. The present study has some limitations that must be considered. The questionnaire on negative life events only addressed five types of events. In addition, the questionnaire was only applied at baseline and, as a result, the authors were unable to monitor the events throughout the subsequent waves. Beyond that, as the questionnaire was self-reported information, the authors cannot rule out a possible memory bias. Moreover, although depression diagnosis was performed in face-to-face interviews using a validated questionnaire, some kind of misclassification is possible, especially in mild cases.

## Conclusion

The present results contribute to the study of the association between negative life events and depression with cross-sectional and prospective data. The association between negative life events and prevalent depression does not reveal many differences between men and women. However, these results show a high burden of negative life events related to incident depression in women compared to men, which needs to be validated in more prospective studies.

## Funding

The ELSA-Brasil received funding from the National Council for Scientific and Technological Development (CNPq) (Wave 1: BA 01 06 0212-00; ES 01 06 0300-00; MG 01 06 0278-00; 01 06 0071-00; RS 01 06 0010-00; SP 01 06 0115-00; Wave 2: BA 01 10 0742-00; ES 01 12 0284-00; MG 01 10 0746-00; RJ 01 11 0093-01; RS 01 10 0643-03; SP 01 10 0773-00; Wave 3: BA 405551/2015-0; ES 405543/2015-8; MG 405552/2015-7; RJ 405544/2015-4: RS 405545/2015-0; SP 405547/2015-3. Simone V. Silva is supported by Coordination of Superior Level Staff Improvement (CAPES) Process: 88887.642866/2021-00, Period: 09/2021-02/2022 and São Paulo Research Support Foundation (FAPESP) Process 2021/09833-1, Period:04/2022-09/2023, with a master scholarship.

## Authors’ contributions

Simone V. Silva: Conceptualization; visualization; formal analysis; data curation; writing; review and editing.

Itamar S. Santos: Formal analysis; writing; review and editing; authorization of the final version for publication.

Danielle B. Lima: Writing; Review and editing; authorization of the final version for publication.

Alessandra C. Goulart: Writing; review and editing, authorization of the final version for publication.

Ana C. Varella: Writing; Review and editing; authorization of the final version for publication.

Paulo A. Lotufo: Data curation; writing; review and editing; authorization of the final version for publication.

André R. Brunoni: Writing; review and editing; authorization of the final version for publication.

Isabela M. Bensenor: Conceptualization; visualization; formal analysis; data curation; writing; review and editing; authorization of the final version for publication.

## Declaration of competing interest

The authors declare no conflicts of interest.
